# Cognitive phenotypes 1 month after ICU discharge in mechanically ventilated patients: a prospective observational cohort study

**DOI:** 10.1186/s13054-020-03334-2

**Published:** 2020-10-21

**Authors:** Sol Fernández-Gonzalo, Guillem Navarra-Ventura, Neus Bacardit, Gemma Gomà Fernández, Candelaria de Haro, Carles Subirà, Josefina López-Aguilar, Rudys Magrans, Leonardo Sarlabous, Jose Aquino Esperanza, Mercè Jodar, Montse Rué, Ana Ochagavía, Diego J. Palao, Rafael Fernández, Lluís Blanch

**Affiliations:** 1 Critical Care Center, Parc Taulí Hospital Universitari, Fundació- I3PT, UAB, Sabadell, Spain; 2 Centro de Investigación Biomédica En Red en Salud Mental (CIBERSAM), Instituto de Salud Carlos III, Madrid, Spain; 3 Department of Clinical and Health Psychology, Universitat Autònoma de Barcelona, Bellaterra, Barcelona, Spain; 4 Centro de Investigación Biomédica En Red en Enfermedades Respiratorias (CIBERES), Instituto de Salud Carlos III, Madrid, Spain; 5 Mental Health Department, Fundació Althaia - Xarxa Assistencial I Universitaria, Manresa, Spain; 6 Critical Care Center, Fundació Althai, Universitat Internacional de Catalunya, Manresa, Spain; 7 Better Care S.L., Barcelona, Spain; 8 Department of Medicine, Universitat de Barcelona, Barcelona, Spain; 9 Neurology Department, Parc Taulí Hospital Universitari, I3PT, UAB, Sabadell, Spain; 10 Departament of Basic Medical Sciences, Universitat de Lleida, Lleida, Spain; 11 Health Services Research Network in Chronic Diseases (REDISSEC), Barcelona, Spain; 12 Mental Health Department, Parc Taulí Hospital Universitari, I3PT, UAB, Sabadel, Spain; 13 Department of Psychiatry and Forensic Medicine, Universitat Autònoma de Barcelona, Bellaterra, Barcelona, Spain

**Keywords:** Cognition in ICU survivors, Neuropsychological profiles, Critical illness, Post-intensive care syndrome

## Abstract

**Background:**

ICU patients undergoing invasive mechanical ventilation experience cognitive decline associated with their critical illness and its management. The early detection of different cognitive phenotypes might reveal the involvement of diverse pathophysiological mechanisms and help to clarify the role of the precipitating and predisposing factors. Our main objective is to identify cognitive phenotypes in critically ill survivors 1 month after ICU discharge using an unsupervised machine learning method, and to contrast them with the classical approach of cognitive impairment assessment. For descriptive purposes, precipitating and predisposing factors for cognitive impairment were explored.

**Methods:**

A total of 156 mechanically ventilated critically ill patients from two medical/surgical ICUs were prospectively studied. Patients with previous cognitive impairment, neurological or psychiatric diagnosis were excluded. Clinical variables were registered during ICU stay, and 100 patients were cognitively assessed 1 month after ICU discharge. The unsupervised machine learning K-means clustering algorithm was applied to detect cognitive phenotypes. Exploratory analyses were used to study precipitating and predisposing factors for cognitive impairment.

**Results:**

K-means testing identified three clusters (K) of patients with different cognitive phenotypes: K1 (*n* = 13), severe cognitive impairment in speed of processing (92%) and executive function (85%); K2 (*n* = 33), moderate-to-severe deficits in learning-memory (55%), memory retrieval (67%), speed of processing (36.4%) and executive function (33.3%); and K3 (*n* = 46), normal cognitive profile in 89% of patients. Using the classical approach, moderate-to-severe cognitive decline was recorded in 47% of patients, while the K-means method accurately classified 85.9%. The descriptive analysis showed significant differences in days (*p* = 0.016) and doses (*p* = 0.039) with opioid treatment in K1 vs. K2 and K3. In K2, there were more women, patients were older and had more comorbidities (*p* = 0.001) than in K1 or K3. Cognitive reserve was significantly (*p* = 0.001) higher in K3 than in K1 or K2.

**Conclusion:**

One month after ICU discharge, three groups of patients with different cognitive phenotypes were identified through an unsupervised machine learning method. This novel approach improved the classical classification of cognitive impairment in ICU survivors. In the exploratory analysis, gender, age and the level of cognitive reserve emerged as relevant predisposing factors for cognitive impairment in ICU patients.

**Trial registration:**

ClinicalTrials.gov Identifier:NCT02390024; March 17,2015.

## Introduction

Cognitive impairment in critically ill survivors who have undergone invasive mechanical ventilation (MV) during the Intensive Care Unit (ICU) stay is a well-established health problem [1–11]. ICU admission is associated with a greater cognitive decline than general ward hospitalization [12], and the rate of dementia diagnosis after 3 years of follow-up has been reported to be higher in elderly ICU survivors than in the general population [13]. This cognitive decline affects the functional and socioeconomic status of ICU survivors and their families, and reduces their quality of life after ICU discharge [5, 14–16]. Cognitive impairment may be as high as 64% in ICU survivors [17]. The domains most commonly affected are attention/concentration, memory and executive function [18].

The pathophysiological mechanisms that lead to cognitive dysfunction after critical illness are not well understood. Many precipitating or modifiable factors and predisposing or non-modifiable factors have been related to the short- and long-term cognitive deficit observed in ICU survivors [19]. Among precipitating factors such as MV [2, 20], length of ICU stay [9, 20], hypoxemia, hypoglycemia, hyperglycemia, fluctuations in serum glucose [20–22] and perceived stress levels during the ICU stay [23], the presence of delirium has shown the closest association with the cognitive impairment observed in ICU survivors [2, 3, 24–26]. Predisposing or non-modifiable factors have been less explored and in most studies have been considered as confounding factors. Nonetheless, older age, previous cognitive impairment and higher illness severity seem to increase the risk of developing cognitive dysfunction after ICU stay [18, 27]. The role of other individual predisposing factors has only rarely been studied in critical illness. One of these factors is cognitive reserve—that is, the ability of the brain to actively address brain dysfunction by using pre-existing cognitive processing approaches or by enlisting compensatory approaches [28]. Cognitive reserve may confer a better resilience to pathological brain changes; that is, people with higher cognitive reserve may be less vulnerable to neurophysiological insults such as the impact of critical illness and its management. Cognitive reserve may also be a target for rehabilitation programs when the brain insult has already occurred.

Most of the studies evaluating cognitive impairment in ICU survivors after MV have focused on global cognitive impairment, and little is known about the characterization of different phenotypes of alteration [12]. The early detection of distinct groups of patients regarding cognitive deficits might reveal the involvement of diverse pathophysiological mechanisms. Thus, the main objective of this study was to describe the cognitive phenotypes 1 month after ICU discharge in survivors of critical illness who had undergone MV during their ICU stay, using an unsupervised machine learning method. Two secondary objectives were also proposed. To warrant clinical interpretation of these results, we contrasted the cognitive phenotypes with the classical definition of cognitive impairment in critically ill patients established by Jackson et al. [7]. An exploratory analysis of the predisposing and precipitating factors (e.g., gender, medications, severity of illness and days with MV) of the cognitive dysfunction after critical care was also carried out.

## Material and methods

### Sample and procedure

This prospective cohort study enrolled patients from two medical/surgical ICUs from October 2015 until December 2017.

The study sample comprised critically ill adult patients (≥ 18 years old) who had undergone invasive MV for at least 24 h during the ICU stay and had been enrolled during the first 48 h of MV. Exclusion criteria were: prior cognitive impairment or dementia, diagnosis of neurological disease, psychiatric disorder, sensory deficits (blindness or deafness), non-Spanish speaking and life expectancy < 3 months. Previous neurological diagnosis (specifically of prior cognitive impairment or dementia) was checked in patients’ clinical records and through interviews with their relatives. In patients > 60 years old, preexisting cognitive impairment was assessed at ICU admission using the Spanish version of the Short Form of the Informant Questionnaire on Cognitive Decline in the Elderly (Short-IQCODE), filled in by relatives [29]. The cutoff point for exclusion on the Short-IQCODE was > 3.56. Daily screening was performed by a critical care nurse. At enrollment, written informed consent was obtained from the patients or their authorized surrogates; if consent was initially obtained from a surrogate, it was subsequently obtained from the patient once s/he was deemed to be mentally competent.

### Measurements

At inclusion, severity of illness was measured by the Acute Physiology and Chronic Health Evaluation (APACHE II) and level of comorbidity by the Charlson Comorbidity Index. Level of consciousness and presence of delirium were assessed daily using the Richmond Agitation Sedation Scale (RASS) and the Confusion Assessment Method for the ICU (CAM-ICU), respectively. Sequential Organ Failure Assessment (SOFA) was measured every day during ICU stay. Number of days receiving sedatives and opioids as well as the accumulated doses of sedation and analgesia were also recorded daily. All sequential data were recorded until ICU discharge or for a maximum of 30 days.

A complete and comprehensive neuropsychological assessment was administered 1 month after ICU discharge. Details of the neuropsychological battery are shown in Table 1. The neuropsychological assessment was administered to all participants by an expert neuropsychologist during a single session lasting between 45 and 60 min. Six cognitive indexes were calculated from the neuropsychological test scores: attention, learning and memory storage, memory retrieval, speed of processing, working memory and executive function.

**Table 1:** Cognitive tests used in the neuropsychological assessment battery

Cognitive domain	Tests
Premorbid intelligence quotient (IQ) estimation	The National Adult Reading Test (NART)—Spanish version- ^S.1^
Verbal attention and working memory	Subtest of Digits from the Wechsler Adult Intelligence Scale version III (WAIS III) ^S.2^
Visual attention and working memory	Subtest of Spatial Span from the Wechsler Memory Scale version III (WMS III) ^S.3^
Learning, short- and long- term verbal memory	Rey Auditory Verbal Learning test ^S.4^
Visual memory	Benton Visual Retention test ^S.5^
Speed of processing	Subtest of Symbol Search (WAIS III) ^S.2^
Speed of processing and Executive function (Automatic response inhibition)	Stroop Color and Word test ^S.6^
Speed of processing and Executive function (Flexibility)	Trail Making Test ^S.7^
Executive function (phonetic verbal fluency)	FAS ^S.8^

See references in the Supplemental Material

### Statistical analysis

Baseline demographics and clinical characteristics during the ICU stay were summarized using medians (IQR) for continuous variables and percentages for categorical variables. For the purposes of analysis, the variable *diagnosis* was operationalized in three categories (medical, surgical and polytrauma). Daily SOFA scores were summarized by a single value defined as the slope of the regression line on the first five SOFA scores. Length of delirium, MV, and sedative and opioid administration were adjusted for days of ICU. This data correction was performed by consensus between authors, on the assumption that data adjusted to the days of ICU can be a better reflection of ICU patients’ true status. However, both variables (with and without adjustment) were initially considered in the univariate analysis. Total doses of opioids (morphine and fentanyl) and sedatives (midazolam, propofol and lorazepam) administered each day were recorded and converted to morphine and midazolam equivalents [30].

The cognitive reserve index was obtained for each patient based on two proxies: educational attainment, defined as the number of years in full-time formal education, and literacy, based on the corresponding intelligence quotient (IQ) score on the Spanish version of the National Adult Reading Test (NART) [31].

Raw scores of the cognitive tests were transformed into z-scores (mean = 0; SD = ± 1) using the normative population data offered by each test. Details of the cognitive index calculation are given in Additional file 1: Table S1.

Two different approaches were used for assessing cognitive impairment in the sample: the first a more classical approach based on the clinical definition of cognitive impairment, and the second a method based on statistical clustering algorithms.

In the classical approach, global cognitive impairment was considered when a patient scored ≥ 1.5 SD below the mean on two or more of the six cognitive indexes, or else when a patient scored ≥ 2 SD below the mean on one or more of the indexes. This clinical definition has been previously used in the literature [7] and is considered a strict, accurate description of cognitive deficit. The z-scores were also used to determine the level of cognitive deficit in each domain for every patient. Likewise, cognitive domain impairment was defined as moderate if the values of the index were ≥ 1.5 SD below the mean and severe if the values were ≥ 2 SD below the mean.

The second approach aimed to find characteristic phenotypes and clusters of patients’ cognitive decline through an unsupervised machine learning technique, the K-means clustering algorithm. K-means clustering is widely used in the data mining field to reveal naturally occurring patterns or groupings, without targeting a specific outcome [32, 33]. The clustering algorithm splits the observations (i.e., patients) into distinct and non-overlapping clusters of cognitive phenotypes based on their cognitive score, without the need to establish an ‘a priori’ definition of cognitive deficit. The procedure was as follows: at each iteration, the algorithm computed the cluster centroids and each observation was assigned to the closest centroid. The Euclidean distance was used as the dissimilarity measure to minimize the within-cluster variation.

The Kruskal–Wallis test was used to analyze the differences between the K groups in the demographic and clinical data, and the Mann–Whitney U test for group comparisons.

Only for exploratory purposes, the association between predisposing and/or precipitating factors and cognitive impairment was studied by binomial logistic regression analyses. Factors with a *p* value < 0.2 in the univariate analysis were included in the multivariable analysis. Modeling adjustments focused on obtaining a simplified model included all variables with *p* values < 0.05 or with clinical relevance for explaining the dependent variable.

## Results

**Fig. 1:**
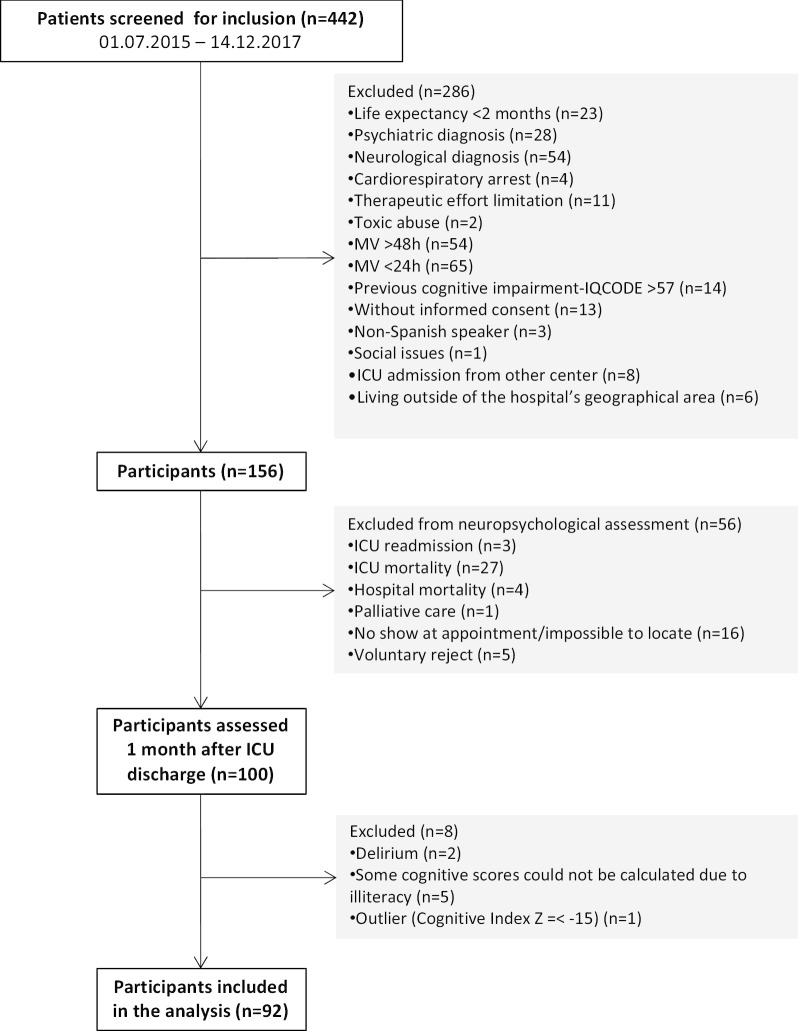
Flowchart representing the distribution of the sample during the different phases of the study

Four hundred and forty-two ICU patients were screened for inclusion. One hundred and fifty-six met the inclusion criteria, of whom 100 were cognitively assessed 1 month after ICU discharge. The final analysis was performed in 92 patients (Fig. 1). Mean age of the sample was 63.03 years (SD 12.78), and 57 participants were male (62%). Further demographic and clinical data of the sample are shown in Table 2.

**Table 2:** Demographic and clinical characteristics of the sample

Demographic and clinical variables	Total sample (N = 92)
Age, yr	64 (56–71)
Female gender	35 (38%)
Cognitive Reserve, z-scores	0.04 (− 0.32 to 0.37)
Diagnosis:	
Medical	74 (80.4%)
Surgical	10 (10.9%)
Polytrauma	8 (8.7%)
APACHE II at ICU admission, points	17 (13–21)
SOFA at ICU admission, points	7 (5–9)
SOFA slope	− 0.9 (− 1.5 to − 0.2)
Charlson Index at ICU admission	3 (2–5)
Length of MV, days	6 (4–10)
MV days ratio	0.64 (0.44–0.77)
Length of delirium, days	1 (0–2)
Delirium ratio	0.037 (0–0.18)
Days with sedatives	4 (2–7)
Days with sedatives ratio	0.37 (0.18–0.58)
Accumulated dose of sedatives, Eq	3.31 (0.95–8.79)
Days with opioids	4 (2–7.75)
Days with opioids ratio	0.37 (0.20–0.62)
Accumulated dose of opioids, Eq	1.02 (0.23–2.44)
Length of ICU stay, days	10.50 (8–16)
Length of hospital stay, days	16.00 (10–34.5)

Data are expressed as n (%) or median (IQR), as appropriate

*IQR* interquartile range, *APACHE* Acute Physiology and Chronic Health Evaluation, *SOFA* Sequential Organ Failure Assessment, *MV* mechanical ventilation, *Eq* equivalents

### Cognitive clusters of patients with the unsupervised machine learning K-means clustering algorithm approach

The K-means test results showed three differential clusters of patients grouped regarding their characteristic cognitive phenotypes (13 patients in K1, 33 in K2 and 46 in K3) (Additional file 1: Fig. S1). The correlation analysis of the composite cognitive variables showed that all cognitive variables could be maintained in the K-means analysis (Additional file 1: Fig. S2).

**Fig. 2:**
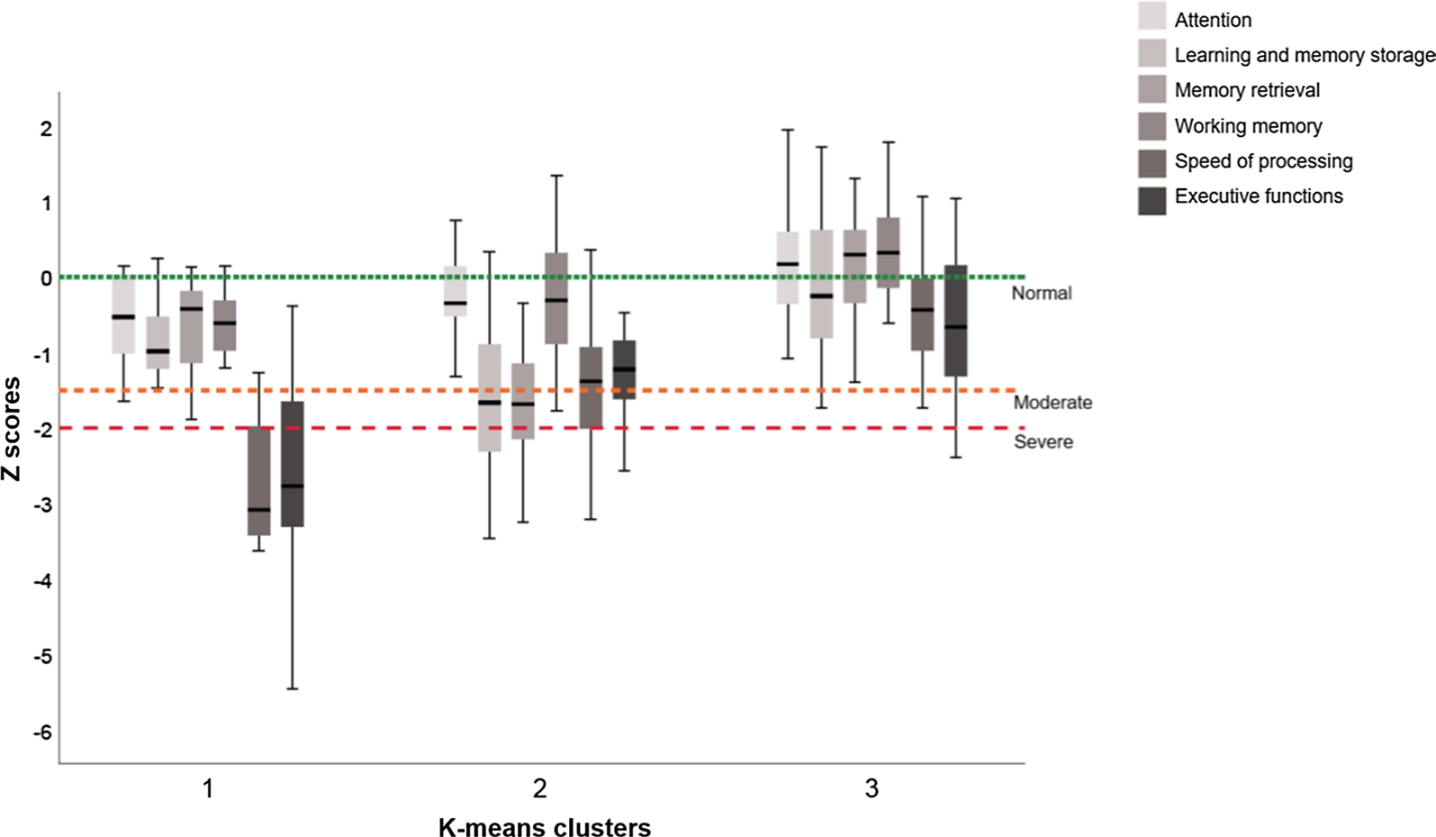
Cognitive function distribution in the three patient clusters. The six cognitive indexes are represented according to each patient cluster. Z-scores between 0 and − 1.5 are considered normal for the cognitive function in question, between − 1.5 and − 2 a moderate deficit, and below − 2 a severe deficit

In the K1 cluster, all patients showed global cognitive impairment: the most altered functions were speed of processing, which affected 93.23% of patients, and executive function, which affected 84.62%. In the K2 cluster, 76% of patients showed global cognitive impairment: 66.67% of participants showed alterations in memory retrieval, 54.5% in learning and memory storage, 36.36% in speed of processing and 33.33% in executive functions. In the K3 cluster, only 11% of patients showed global cognitive impairment: 15.22% presented impaired executive function and 13.04% reduced speed of processing. Further details are given in Fig. 2 and in Additional file 1: Table S2.

### Prevalence of global cognitive impairment and cognitive domain deficits using the classical approach: comparison with the K-means clustering algorithm approach

Using the classical approach, 43 patients (47%) presented global cognitive impairment. When the six cognitive indexes were explored separately, the most commonly affected domain was speed of processing (33 patients, 36%), followed by executive function (29 patients, 31%), memory retrieval (23 patients, 25%) and learning and memory storage ability (20 patients, 22%). Attention and working memory were marginally affected, with one patient (1%) showing a deficit in each domain. Note that the same patient could present deficits in different cognitive domains at the same time.

**Fig. 3:**
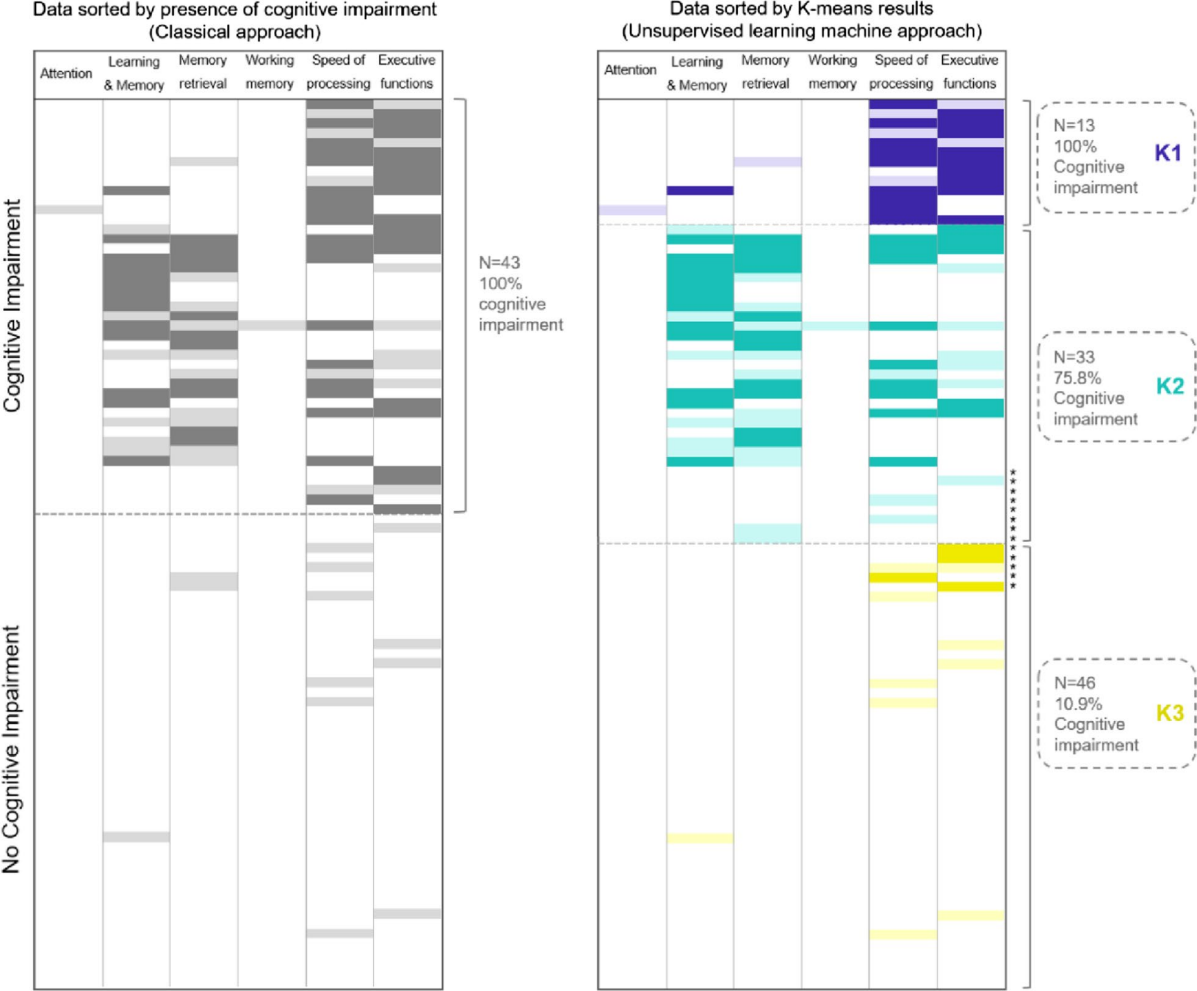
Comparison of patients with the two approaches used to analyze global cognitive impairment. The classical approach identifies patients with and without global cognitive impairment, while the unsupervised learning machine method also determines specific cognitive profiles. With the classical definition of global cognitive impairment, the k-means test classified five patients with global cognitive deficit in the K3 cluster, a group in which most patients presented a non-cognitive deficit profile. Moreover, eight patients without global cognitive impairment were grouped in K2, a cluster with a greater presence of patients with cognitive impairment. *Patients incorrectly classified by the unsupervised learning machine approach according to the classical definition of global cognitive impairment

The results obtained with the two approaches were compared in order to warrant a feasible clinical interpretation of the groups obtained through the unsupervised machine learning approach. Compared to the classical definition of cognitive impairment, the K-means test classified most of the participants with cognitive impairment between the K1 and K2 groups; however, 89% of the participants in K3 did not present cognitive impairment. Thus, the K-means test failed to classify 13 patients (14.1%) but correctly classified 79 (85.9%) (Fig. 3).

### Analysis of predisposing and precipitating factors

The descriptive analysis of the differences in the demographic and clinical variables between the three phenotypes showed that in K2 there were more women (*p* = 0.001), patients were older (*p* < 0.001) and had more comorbidities (*p* < 0.001) than in K1 or K3. Significant differences were also found in days (*p* = 0.016) and doses (*p* = 0.039) of opioid treatment in K1 than in K2 or K3. Cognitive reserve was significantly higher in K3 (*p* = 0.001) than in K1 or K2. Further details of the demographic and clinical data according to cluster group and percentage of diagnosis are shown in Table 3 and in Additional file 1: S3.

**Table 3:** Demographic and clinical characteristics of the sample according to cluster group

Demographic and clinical variables	K1 (N = 13)	K2 (N = 33)	K3 (N = 46)	P(< 0.05)
*Age, years*^*a,c*^	59 (51.5–63.5)	72 (66–78)	60 (50–69)	* < 0.001*
*Gender (%)*				
Female gender	3 (23.1)	21 (63.6)	11 (23.9)	*0.001*
*Cognitive reserve*^*b,c*^	− 0.07 (− 0.94 to − 0.05)	− 0.11 (− 0.45 to − 0.23)	0.31 (− 0.08 to 0.68)	* < 0.001*
Diagnosis (%)				0.507
Medical	12 (92.3)	27 (81.8)	35 (76.1)	
Surgical	1 (7.7)	5 (15.2)	4 (8.7)	
Polytrauma	0 (0.00)	1 (3.0)	7 (15.2)	
APACHE II at ICU admission, points	15 (12–18)	18 (15.5–22.5)	17 (12–20.25)	0.082
SOFA at ICU admission, points	8 (5.5–10.5)	7 (4–10)	7 (5–9)	0.719
SOFA slope	− 1.1 (− 1.8 to − 0.35)	− 0.9 (− 1.5 to − 0.25)	− 0.75 (− 1.4 to − 0.12)	0.536
*Charlson Index at ICU admission*^*a,c*^	3 (2.5–4)	5 (3–6)	3 (1–4)	* < 0.001*
Length of MV, days	5 (4.5–10)	5 (3–8)	7 (4–11)	0.222
MV days ratio	0.67 (0.45–0.73)	0.64 (0.38–0.71)	0.63 (0.44–0.8)	0.492
Length of delirium, days	0 (0–1)	1 (0–2)	0 (0–2)	0.451
Delirium ratio	0 (0–0.12)	0.08 (0–0.22)	0 (0–0.10)	0.367
*Accumulated dose of sedatives*^*c*^	4.29 (0.29–16.38)	1.35 (0.35–6.20)	3.85 (1.92–10.62)	*0.050*
*Accumulated dose of opioids*^*b*^	0.00 (0.00–2.33)	0.83 (0.29–2.10)	1.45 (0.63–2.52)	*0.039*
Days with sedatives	4 (1–7)	3 (1–6.5)	4 (3–7.25)	0.178
Days with sedatives ratio	0.44 (0.13–0.64)	0.28 (0.12–0.47)	0.40 (0.22–0.60)	0.163
*Days with opioids*^*a,b*^	0 (0–5)	4 (2–7)	5 (3–9)	*0.016*
*Days with opioids ratio*^*a,b*^	0.00 (0.00–0.37)	0.35 (0.22–0.61)	0.5 (0.33–0.64)	*0.007*
Length of ICU stay, days	11 (7–12.5)	9 (7–18.5)	11 (8–16.25)	0.604
Length of hospital stay after ICU discharge, days	15 (9–30)	17 (11–49)	17.50 (9–31.5)	0.591

Data are expressed as n (%) or median (IQR), as appropriate. *IQR* interquartile range, *APACHE* Acute Physiology and Chronic Health Evaluation, *SOFA* Sequential Organ Failure Assessment, *MV* mechanical ventilation

^a^Significant difference between K1 versus K2

^b^Significant difference between K1 versus K3

^c^Significant difference between K2 versus K3

Since the K1 cluster contained only 13 patients, it was merged in a single group with the K2 cluster in order to explore the influence of predisposing and precipitating factors on global cognitive impairment by means of the binomial logistic regression model. The exploratory analysis was run in a group (K1 + K2) in which most participants were cognitively impaired versus the group (K3) in which most participants had a normal cognitive status. All variables from the univariate analyses with a *p* value < 0.2 (Additional file 1: Table S4) were included in a multivariable model. A reduced final multivariable model was run including the significant factors (gender, cognitive reserve and days with opioids) from the initial multivariable model and ‘age’ as a confounding factor. Age (OR 1.05; 95% CI 1.00–1.01; *p* = 0.048), gender (OR 2.81; 95% CI 1.01–7.84; *p* = 0.048) and cognitive reserve (OR 0.37; 95% CI 0.16–0.83; *p* = 0.016) resulted statically significant, while days with opioids ratio presented a trend toward significance (OR 0.17; 95% CI 0.03–1.08; *p* = 0.061). Further details of the multivariable regression models are shown in Additional file 1: Table S5. The receiver operating characteristic (ROC) curve analysis of the final multivariable regression model is shown in Additional file 1: Figure S3.

## Discussion

The main finding of this study is the characterization of three different cognitive phenotypes in critically ill-ventilated patients 1 month after ICU discharge using the unsupervised machine learning K-means clustering algorithm. The descriptive and exploratory analysis of factors revealed female gender and older age as potential risk factors for specific cognitive impairment, while cognitive reserve emerged as a protective factor against cognitive deficit.

This is the first study to report patterns of cognitive impairment after ICU discharge in mechanically ventilated patients based on an unsupervised machine learning algorithm clustering method. This novel approach allowed us to detect three different phenotypes in the ICU survivors based on the exploration of six cognitive domains, instead of the two categories (impaired vs. non-impaired) differentiated in the classical method by Jackson et al. [7]. Furthermore, in two of the three phenotypes, different levels and types of cognitive impairment were observed in the participants: while the K1 phenotype was characterized by severe alterations in speed of processing and executive function, the K2 phenotype was distinguished by moderate-to-severe deficits in learning and memory retrieval, and impaired speed of processing and executive function. The last phenotype (K3) was characterized by an almost normal performance in most participants on most of the cognitive domains assessed. These results are in line with other studies [5, 8, 9], except for the low impairment in attention.

The presence of cognitive alterations in different domains in ICU survivors might suggest different patterns of brain dysfunction which probably involve various pathophysiological mechanisms or pathways. More specifically, while speed of processing, executive functions and memory retrieval impairments are related to dysfunctions in subcortical areas and the frontal lobe, the presence of learning and memory storage problems points to alterations in the hippocampus and the medial temporal lobe.

Using the classical approach for cognitive impairment, almost half of the participants (47%) showed moderate-to-severe cognitive impairment 1 month after ICU discharge. This low incidence in comparison with other studies [2, 3, 7] may be related to the patients’ clinical characteristics, given that our sample presented lower severity of illness and shorter duration of delirium during ICU stay than samples in other reports. Moreover, while pre-existing cognitive impairment was ruled out in our patients, in other studies it may have contributed to the overall cognitive impairment observed. Interestingly, when the results of the unsupervised learning machine method were compared with the classical approach, 86% of the participants were accurately classified between the two categories of impaired and non-impaired cognition. Thus, the unsupervised learning machine method not only allowed detection of cognitive decline but also improved the classification of patients by identifying different patterns of cognitive impairment among ICU survivors.

The three cognitive phenotypes differed in terms of several demographic and clinical factors, a circumstance that may have had an impact on how the clusters were configured. Patients with the K1 phenotype had significantly fewer days of opioid treatment than patients in K2 and K3, and lower accumulated doses of opioids than K3. Participants in the K2 phenotype were mostly women, older and had more comorbidities than those in K1 and K3. Moreover, they presented lower accumulated doses of sedative than K3. Finally, participants with the K3 phenotype showed higher levels of cognitive reserve than K1 and K2.

When the most cognitively affected phenotypes were combined (K1 and K2), the exploratory analysis of the predisposing and precipitating factors suggested that certain factors may play a more important role than others for the development of cognitive decline after ICU. Specifically, female gender, older age and a lower level of cognitive reserve were significantly associated with cognitive impairment.

Looking at these factors individually, we found that women were more likely to present cognitive impairment 1 month after ICU discharge. The role of gender in the cognitive impairment after ICU has not been specifically addressed, and the occasional references in the literature are contradictory [5, 34, 35]. Our results coincide with Habib et al. [35] in suggesting that female gender may be a risk factor for developing post-ICU cognitive impairment, at least early after ICU discharge. However, these conclusions should be interpreted with caution: although in healthy populations older women usually perform better in verbal memory tests than older men [36], normal aging itself entails cognitive deficits, especially memory and speed of processing [37]. Previous results for the impact of age on the cognitive status of ICU survivors are controversial [3, 4, 9, 25, 34, 35, 38]. Our results corroborate the notion that older critical care patients are more likely to present cognitive decline after critical illness. However, we cannot rule out a relation between age and gender in our sample, since patients in phenotype K2 (deficits in memory, speed of processing and executive dysfunction) tended to be female, older and presented more comorbidities. If female patients are commonly older, and if aging affects cognitive status in women differently than in men, it may be that the impact of ICU stay on cognition in older critically ill patients also differs between genders.

In this study, patients in the phenotype with the best cognitive performance (K3) presented the highest level of cognitive reserve. Although the analysis is only exploratory, cognitive reserve was found to be a protective factor against cognitive alterations 1 month after ICU survival. Only one previous study [10] has included this concept as a predisposing factor for cognitive decline in ICU patients. Interestingly, cognitive reserve has also been identified as a protective factor for cognitive decline in healthy older adults [39, 40] and in a wide range of medical populations [41–43], including older patients with postsurgical delirium [44].

One of the phenotypes with cognitive impairment (K1) presented significantly fewer days with opioid treatment than the others. Although the ratio between days with opioids and days of ICU stay only reached a trend towards significance in the exploratory analysis, opioid treatment was the only precipitating factor that could be related to cognitive decline in our sample. It should be borne in mind that opioid treatment improves the welfare and comfort of critically ill patients, enhancing their emotional status. This emotional well-being related to the management of the analgesia and sedation during ICU stay may impact the cognitive status of survivors.

Duration of delirium was not related to cognitive impairment. This may have been due to the notably short duration of delirium in our patients and the low inter-subject variability; furthermore, our patients were assessed 1 month after ICU discharge, while in the other studies the impact of delirium on cognition was assessed at 3 or 12 months.

Although the analysis was only exploratory, our results suggested a higher burden of predisposing factors (such as gender, age and cognitive reserve) than precipitating factors in the specific cognitive impairment detected early after ICU discharge.

The current results should be confirmed in future studies with a higher number of participants. Nevertheless, our preliminary findings may serve as a starting-point for further research. Of particular interest is the evolution of the cognitive sequelae in the two phenotypes with cognitive impairment. Establishing how patients in K1 and K2 resolve (or maintain) their cognitive deficits might help to clarify the burden of predisposing factors in long-term cognitive decline in ICU survivors. Determining how brain changes associated with aging in both genders may be impacted by critical illness, and how cognitive reserve may decrease this impact, also deserves further investigation.

The main limitation of this study is the size of the sample obtained in one of the clusters generated by the K-means clustering algorithm. This small size hampered the analysis of the role of the predisposing and precipitating factors in the three cognitive phenotypes in the ICU MV survivors. The two phenotypes that included most of their participants with cognitive impairment had to be combined in a single group in order to run the analysis. Nonetheless, the analysis was underpowered and it must be considered as exploratory. However, the strict selection of participants (with control of any previous cognitive impairment), and the careful statistical analysis vouch for the accuracy of our conclusions. The optimal interval for cognitive assessment may also be a limitation for performing comparisons with other studies, although it was appropriate for detecting the cognitive phenotype in the early stages of the recovery phase.

## Conclusions

Three cognitive phenotypes were identified in the critically ill mechanically ventilated survivors 1 month after ICU discharge using the unsupervised machine learning K-means clustering algorithm. This approach improved the classical classification of patients by identifying different patterns of cognitive impairment among ICU survivors. Despite the exploratory nature of the analysis, female gender, older age and low cognitive reserve seemed to play relevant roles as predisposing factors for severe cognitive impairment in these patients. It is worth noting that the findings of the factors’ analysis are statistically underpowered, and that further studies should be conducting to obtain definite conclusions. Thus, critical illness, together with the predisposing characteristics of each patient, might trigger different brain dysfunctions the mechanically ventilated patients at early stages of the ICU recovery.

## Supplementary information

**Additional file 1:** This additional file contains Tables S1–S5, supplemental references and Figures S1–S3.

## Data Availability

The datasets used and analyzed during this study are available from the corresponding author on reasonable request.
