# Supplementary material for: Cognitive phenotypes 1 month after ICU discharge in mechanically ventilated patients: a prospective observational cohort study
Source: Crit Care. 2020 Oct 21;24:618. doi: 10.1186/s13054-020-03334-2 (PMC7579874; doi:10.1186/s13054-020-03334-2)
Supplement: Supplementary file 1 — Additional file 1: This additional file contains Tables S1–S5, supplemental references and Figures S1–S3. [file 13054_2020_3334_MOESM1_ESM.docx]

**Supplemental Material**

Article title: **Cognitive phenotypes one month after ICU discharge in mechanically ventilated patients: a prospective observational cohort study**

Authors: Sol Fernández-Gonzalo*; Guillem Navarra-Ventura; Neus Bacardit ; Gemma Gomà Fernández; Candelaria De Haro C; Carles Subirà; Josefina López Aguilar; Rudys Magrans; Leonardo Sarlabous; Jose Aquino Esperanza ; Mercè Jodar; Montse Rué; Ana R. Ochagavía; Diego J. Palao; Rafael Fernández; Lluís Blanch

*Corresponding Author Information

Sol Fernández-Gonzalo

Critical Care Center, Parc Taulí Hospital Universitari, I3PT, CIBERSAM, UAB, Spain

[msfernandez@tauli.cat](mailto:msfernandez@tauli.cat)

**Table S1** Cognitive index calculation

| **COGNITIVE INDEXES** | **COGNITIVE TESTS & FORMULA** |
| --- | --- |
| **Attention** | (Digit forward subtest + Spatial forward Span)/2 |
| **Learning and memory storage** | (Number of words learned on the Rey Auditory Verbal Learning Test + Number of words remembered at long term)/2 |
| **Memory retrieval** | (Nº of words retrieval at long term + nº of correct answers in Benton visual recognition test)/2 |
| **Speed of processing** | (Symbol search score + TMT A + Number of words read in Stroop test)/3 |
| **Working Memory** | (Digit backward subtest + Spatial backward Span)/2 |
| **Executive Function** | (Trail Making Test B-A + Number of word-color read in Stroop Test + phonetic verbal fluency)/3 |

**Table S1** The calculation of the six cognitive indexes was based on the different subtests of the neuropsychological assessment. All indexes are presented as Z-scores (mean=0, SD= ±1). Z-scores were age-adjusted and, when possible, gender- and education-adjusted, using the normative population data offered for each cognitive test norm.

**Table S2** Level of impairment of each function by cluster

| **COGNITIVE INDEX** | **LEVEL OF IMPAIRMENT**  ^a,b^ | **K1 (N=13)** | | **K2 (N=33)** | | **K3 (N=46)** | | **TOTAL SAMPLE (N=92)** | | | |
| --- | --- | --- | --- | --- | --- | --- | --- | --- | --- | --- | --- |
|  |  | **N** | **%** | **N** | **%** | **N** | **%** | **N** | **%** | **N** | **%** |
| Attention | Moderate | 1 | 7.7 | 0 | 0.0 | 0 | 0.0 | 1 | 1.1 | 1 | 1.1 |
|  | Severe | 0 | 0.0 | 0 | 0.0 | 0 | 0.0 | 0 | 0.0 |  |  |
| Working memory | Moderate | 0 | 0.0 | 1 | 3.0 | 0 | 0.0 | 1 | 1.1 | 1 | 1.1 |
|  | Severe | 0 | 0.0 | 0 | 0.0 | 0 | 0.0 | 0 | 0.0 |  |  |
| Learning | Moderate | 0 | 0.0 | 6 | 18.2 | 1 | 2.2 | 7 | 7.6 | 20 | 21.7 |
|  | Severe | 1 | 7.7 | 12 | 36.4 | 0 | 0.0 | 13 | 14.1 |  |  |
| Memory retrieval | Moderate | 1 | 7.7 | 11 | 33.3 | 0 | 0.0 | 12 | 13.0 | 23 | 25.0 |
|  | Severe | 0 | 0.0 | 11 | 33.3 | 0 | 0.0 | 11 | 11.9 |  |  |
| Speed of processing | Moderate | 3 | 23.2 | 3 | 9.1 | 5 | 10.9 | 11 | 11.9 | 33 | 35.9 |
|  | Severe | 9 | 69.2 | 9 | 27.3 | 1 | 2.2 | 22 | 23.9 |  |  |
| Executive function | Moderate | 2 | 15.4 | 5 | 15.2 | 4 | 8.7 | 11 | 11.9 | 29 | 31.5 |
|  | Severe | 9 | 69.2 | 6 | 18.2 | 3 | 6.5 | 28 | 19.6 |  |  |

**Table S2** Number of patients and percentage of moderate and severe cognitive dysfunction in the six cognitive domains.

^a^ Moderate impairment= participants with z- score <-1.5 and -2.

^b^ Severe impairment= participants with z-score <-2

**Table S3** Diagnosis at ICU admission in each cluster

| **DIAGNOSIS** | **K1 (N=13)** | | **K2 (N=33)** | | **K3 (N=46)** | | **TOTAL SAMPLE (N=92)** | |
| --- | --- | --- | --- | --- | --- | --- | --- | --- |
|  | N | % | N | % | N | % | N | % |
| Sepsis | 2 | 15.4 | 9 | 27.3 | 13 | 28.3 | 24 | 26.1 |
| Pneumonia | 1 | 7.7 | 3 | 9.1 | 10 | 21.7 | 14 | 15.2 |
| Metabolic | 0 | 0.0 | 1 | 3.0 | 2 | 4.3 | 3 | 3.3 |
| Polytrauma | 0 | 0.0 | 1 | 3.0 | 7 | 15.2 | 8 | 8.7 |
| Postsurgical | 1 | 7.7 | 5 | 15.1 | 4 | 8.7 | 10 | 10.9 |
| Acute respiratory insufficiency | 3 | 23.1 | 2 | 6.1 | 6 | 13.0 | 11 | 12.0 |
| Heart failure | 1 | 7.7 | 3 | 9.1 | 1 | 2.2 | 5 | 5.4 |
| Cardiorespiratory arrest | 2 | 15.4 | 2 | 3.0 | 1 | 2.2 | 4 | 4.3 |
| Other causes (e.g., pancreatitis, gastrointestinal hemorrhage…) | 3 | 23.1 | 8 | 24.2 | 2 | 4.3 | 13 | 14.1 |

**Table S3** Type and percentage of each diagnosis are shown by clusters and in the total sample

**Table S4** Univariate logistic regression analyses

| **Factors** | | **OR** | **95% CI** | **SE** | **p** |
| --- | --- | --- | --- | --- | --- |
| Predisposing factors | **Age, yr ^a^** | 1.07 | (0.07 - 1.12) | 0.02 | **0.001** |
|  | **Gender  ^a^** | 3.48 | (1.42- 8.57) | 0.46 | **0.006** |
|  | **Cognitive Reserve  ^a^** | 0.27 | (0.12 - 0.59) | 0.4 | **0.001** |
|  | **APACHE II at ICU admission, points ^a^** | 1.05 | (0.98 - 1.12) | 0.04 | **0.159** |
|  | SOFA at ICU admission, points | 1.02 | (0.89 - 1.16) | 0.07 | 0.802 |
|  | **Charlson Index at ICU admission ^a^** | 1.34 | (1.10 - 1.64) | 0.1 | **0.004** |
|  | **Diagnosis  ^a^** | 0.13 | (0.02 - 1.13) | 1.1 | **0.064** |
|  | ------------------------------------------------ |  |  |  |  |
| Precipitating factors | SOFA slope | 0.82 | (0.47 - 1.43) | 0.28 | 0.481 |
|  | Length of MV, days | 0.98 | (0.95 - 1.02) | 0.02 | 0.393 |
|  | MV days ratio | 0.40 | (0.06 - 2.54 | 0.94 | 0.333 |
|  | Length of delirium, days | 1.08 | (0.84 - 1.38) | 0.13 | 0.559 |
|  | Delirium ratio | 2.90 | (0.25 - 33.31) | 1.25 | 0.393 |
|  | Accumulated dose of sedatives | 0.99 | (0.95 - 1.02) | 0.02 | 0.535 |
|  | Accumulated dose of opioids | 0.91 | (0.79 - 1.05) | 0.07 | 0.205 |
|  | Days with sedatives | 0.95 | (0.86 - 1.04) | 0.05 | 0.244 |
|  | **Days with sedatives ratio  ^a^** | 0.26 | (0.05 - 1.43) | 0.86 | **0.083** |
|  | Days with opioids | 0.95 | (0.88 - 1.03) | 0.04 | 0.221 |
|  | **Days with opioids ratio  ^a^** | 0.15 | (0.03 - 0.76) | 0.82 | **0.022** |
|  | Length of ICU stay, days | 0.99 | (0.96 - 1.02) | 0.01 | 0.512 |
|  | Length of hospital stay after ICU discharge, days | 1.01 | (0.98 - 1.01) | 0.01 | 0.478 |

**Table S4** Results of the screening analyses for the selection of variables in the multivariable analysis. Abbreviations: APACHE= Acute Physiology and Chronic Health Evaluation; SOFA= Sequential Organ Failure Assessment; MV= Mechanical Ventilation; OR= Odd Ratio; CI= Confidence Interval; SE= Standard Error

^a^ P Value<0.2

**Table S5** Multivariable regression models

| Initial multivariable regression model | | | | | |
| --- | --- | --- | --- | --- | --- |
| **Factors** | **OR** | **95% CI** | **SE** | **p** | **VIF** |
| Age, yr | 1.04 | (0.96 - 1.11) | 0.04 | 0.332 | 2.24 |
| **Gender** | 3.62 | (1.13- 11.57) | 0.59 | **0.030** | 1.32 |
| **Cognitive reserve** | 0.38 | (0.16 - 0.90) | 0.44 | **0.027** | 1.15 |
| APACHE II at ICU admission, points | 1.01 | (0.93 - 1.10) | 0.04 | 0.765 | 1.11 |
| Charlson Index at ICU admission | 1.10 | (0.78 - 1.55) | 0.18 | 0.586 | 2.46 |
| Diagnosis | 0.63 | (0.04 - 8.74) | 1.35 | 0.727 | 1.13 |
| Days with sedatives ratio | 3.42 | (0.31 - 37.56) | 1.23 | 0.315 | 1.57 |
| **Days with opioids ratio** | 0.11 | (0.01 - 0.94) | 1.09 | **0.043** | 1.40 |
|  | | | | | |
| Reduced multivariable regression model | | | | | |
| **Factors** | **OR** | **95% CI** | **SE** | **p** | **VIF** |
| **Age, yr** | 1.05 | (1.00 - 1.10) | 0.024 | **0.048** | 1.05 |
| **Gender** | 2.81 | (1.01 - 7.84) | 0.520 | **0.048** | 1.03 |
| **Cognitive reserve** | 0.37 | (0.16 - 0.83) | 0.416 | **0.016** | 1.03 |
| Days with opioids ratio | 0.17 | (0.03 - 1.08) | 0.932 | 0.061 | 1.02 |

**Table S5** The initial multivariable regression model included all factors that were significant (p<0.2) in the univariate model. Although ‘age’ showed a p value >0.05, it was maintained in the adjusted multivariable model as it was considered as a confounding factor for cognitive impairment. A final multivariable model was run including the statistically significant factors (p<0.05) from the initial multivariable model and ‘age’ as a confounding factor for cognitive impairment. Multicollinearity analysis of variables was tested using the Variance Inflation Factor (VIF).

**References (Supplemental Material)**

S.1. Del Ser T, González-Montalvo JI, Martínez-Espinosa S, et al (1997) Estimation of premorbid intelligence in Spanish people with the Word Accentuation Test and its application to the diagnosis of dementia. Brain Cogn 33:343–356

S.2. Wechsler D (1999) Escala de inteligencia Wechsler para Adultos (WAIS-III manual). TEA. Madrid

S.3. Wechsler D (2004) Wechsler Memory Scale III (WMS-III manual). TEA. Madrid

S.4. Rey A (1964) L’examen clinique en psychologie [The clinical psychological examination]. Universitaire de France, Paris

S.5. Benton A (1974) The Revise Visual Retention Test. 4th ed. Psychological Corporation, New York

S.6. Golden C, Freshwatere S (2002) The Stroop Color and Word Test: A Manual for Clinical and Experimental Uses. Stoelting, Chicago

S.7. Reitan R, Wolfson D (1985) The Halstead-Reitan Neuropsychological Test Battery. Clinical Neuropsychological Press, Tucson

S.8. Artiola i Fortuny L, Hermosillo Romo D, Heaton R, et al (1999) Manual de normas y procedimientos para la batería neuropsicológica en español. M Press, Tucson

**Figure S1** Clusters of participants based on K-means clustering.


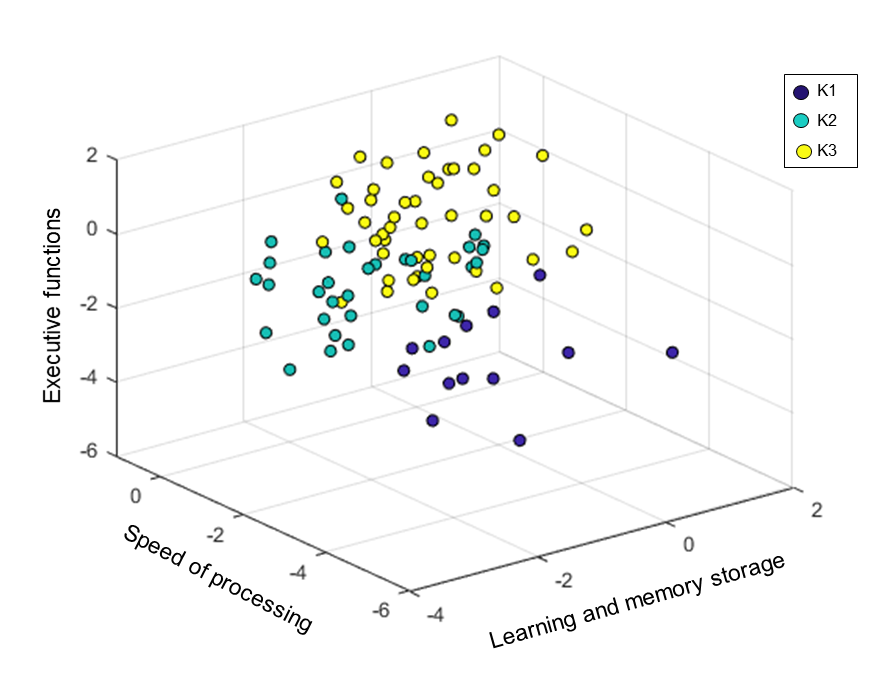


**Fig. S1.** Spatial representation using three of the six variables considered in the K-means analysis. The selection of the three variables displayed is arbitrary.

**Figure S2**. Correlation matrix of the composite cognitive indexes


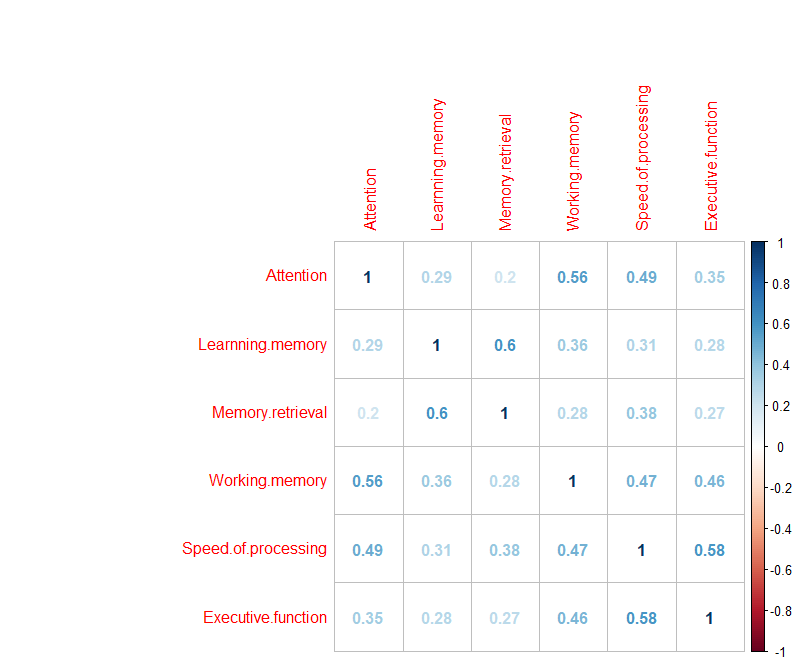


**Fig. S2.** Correlation matrix of the composite cognitive variables using the Spearman correlation index. As observed, moderate correlations were found between attention and working memory index (0.56), between memory retrieval and learning memory (0.6), and between executive function and speed of processing (0.58). These moderate correlations are not strong enough to exclude any of the variables involved in the composite cognitive index.

**Figure S3.** Receiver Operating Characteristic (ROC) curve analysis of the reduced multivariable analysis

**
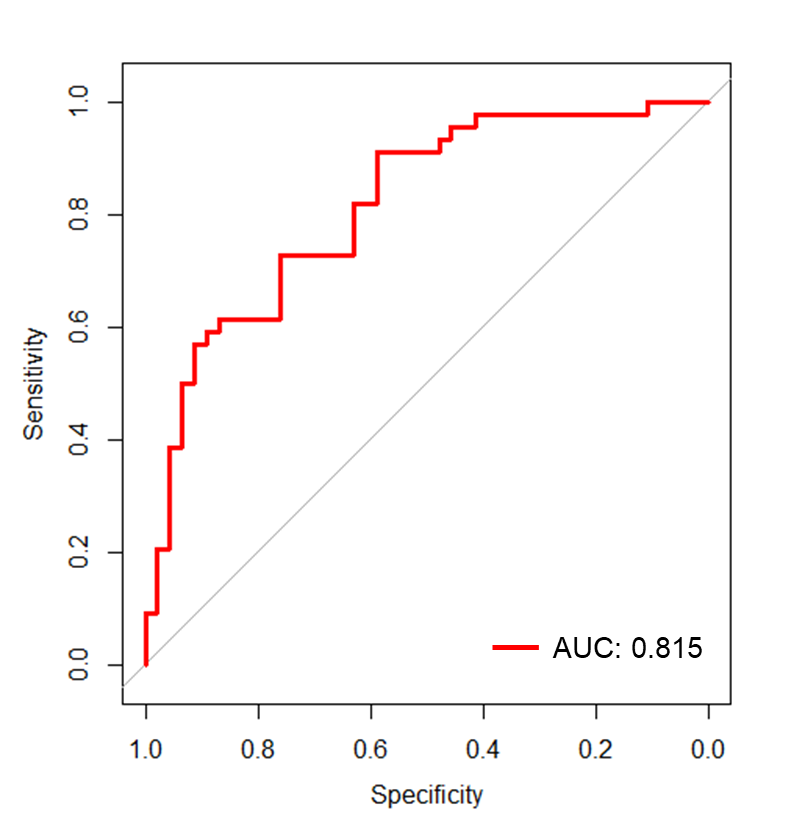
**

**Fig. S3** Discriminant power data of the adjusted multivariable model.
